# The Use of ^68^Ga-DOTATATE PET/CT in the Non-invasive Diagnosis of Optic Nerve Sheath Meningioma: A Case Report

**DOI:** 10.3389/fonc.2018.00454

**Published:** 2018-10-16

**Authors:** Karine A. Al Feghali, Debra N. Yeboa, Beth Chasen, Maria K. Gule, Jason M. Johnson, Caroline Chung

**Affiliations:** ^1^Division of Radiation Oncology, Department of Radiation Oncology, University of Texas MD Anderson Cancer Center, Houston, TX, United States; ^2^Division of Diagnostic Imaging, Department of Nuclear Medicine, University of Texas MD Anderson Cancer Center, Houston, TX, United States; ^3^Division of Diagnostic Imaging, Department of Diagnostic Radiology, University of Texas MD Anderson Cancer Center, Houston, TX, United States

**Keywords:** optic nerve sheath meningioma, positron-emission tomography and computed tomography, ^68^Ga-DOTATATE PET/CT, orbital neoplasms, meningioma, somatostatin (analogs and derivatives)

## Abstract

We hereby report the case of a patient with optic nerve sheath meningioma (ONSM), whose diagnosis and multidisciplinary management was guided by the use of Gallium-68 (^68^Ga)-labeled dodecanetetraacetic acid-tyrosine-3-octreotate (DOTATATE) positron emission tomography (PET)/computed tomography (CT) scan. We briefly review the diagnosis and management of ONSM, and review the literature on the role and current status of nuclear imaging with somatostatin receptor ligands in the non-invasive diagnosis and management of meningiomas.

## Introduction

Orbital space-occupying lesions include a wide variety of benign and malignant diseases, including orbital inflammatory pseudotumor, optic nerve sheath meningioma (ONSM), orbital metastases, orbital lymphoma, and optic pathway gliomas (1). ONSMs represent 2% of orbital tumors (2, 3) and 1–2% of all intracranial meningiomas (3). Different diagnostic strategies have been employed to aid in the diagnosis of orbital tumors. The main imaging modalities used currently to aid in diagnosis include contrast-enhanced computed tomography (CT) and magnetic resonance imaging (MRI) (4). However, in certain circumstances, these imaging modalities are not sufficient to make a definitive diagnosis, and alternative methods are needed to avoid an invasive open orbital biopsy.

This is one of the few reported cases on the use of Gallium-68 (^68^Ga)-labeled dodecanetetraacetic acid-tyrosine-3-octreotate (DOTATATE) positron emission tomography (PET)/CT scan for non-invasive diagnosis of an orbital space-occupying tumor.

## Case presentation

A 28-year old female presented with a history of progressive left-sided temporal vision loss over a year. She noticed that she was running into objects and people on the left side of her field of vision. The patient also complained of a dull ache in her left eye but denied any other focal neurological symptoms.

On physical examination, the only pertinent finding was left temporal hemianopia.

**Figure 1:**
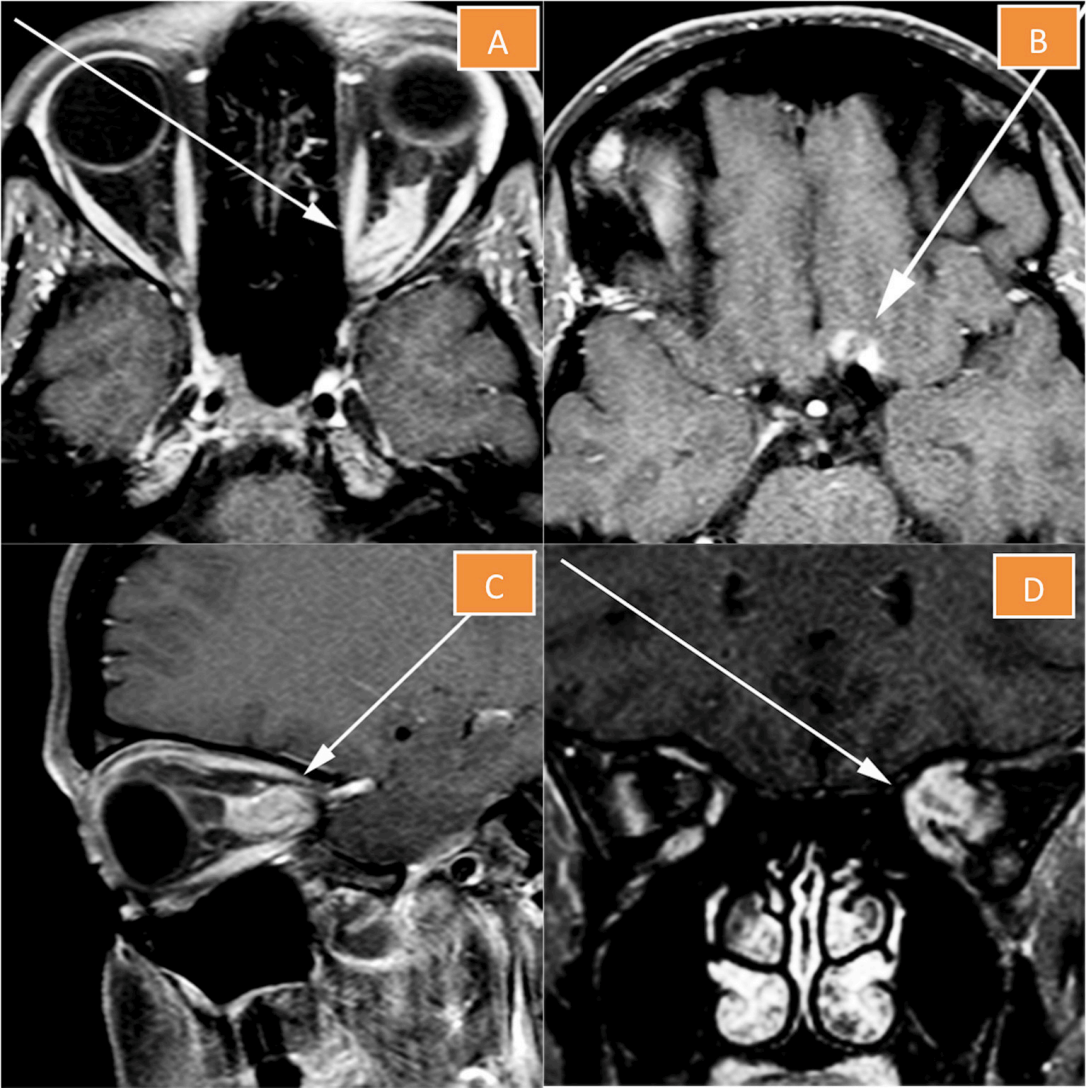
Magnetic resonance imaging of the left orbit. The lesion essentially replaces the optic nerve **(A,C,D)**, and has a component extending superiorly onto the left side of the planum sphenoidale **(B)**.

MRI of the orbits with contrast revealed a heterogeneously enhancing large mass lesion occupying the mid- and posterior thirds of the optic nerve pathway. The typical “tram-track” appearance of sheath enhancement around the central optic nerve expected for an optic nerve meningioma was absent. Rather, the lesion essentially replaced the optic nerve and appeared to demonstrate infiltration into the nerve, which raised the suspicion for an optic nerve glioma (Figure 1). The lesion extended through the optic canal with a component extending superiorly onto the left side of the planum sphenoidale rather than remaining intrinsic to the nerve, as it would be expected for an optic nerve glioma. By virtue of dural involvement of the planum sphenoidale, this mass was suggestive of an atypical left ONSM, which had on some images apparently replaced the nerve (Figure 1). Given this unusual clinical presentation and the lack of typical findings on MRI, decision was taken in a multidisciplinary tumor board to proceed with a ^68^Ga-DOTATATE PET/CT scan, particularly to rule out an optic nerve glioma. Biopsy was deemed too morbid in this context.

**Figure 2:**
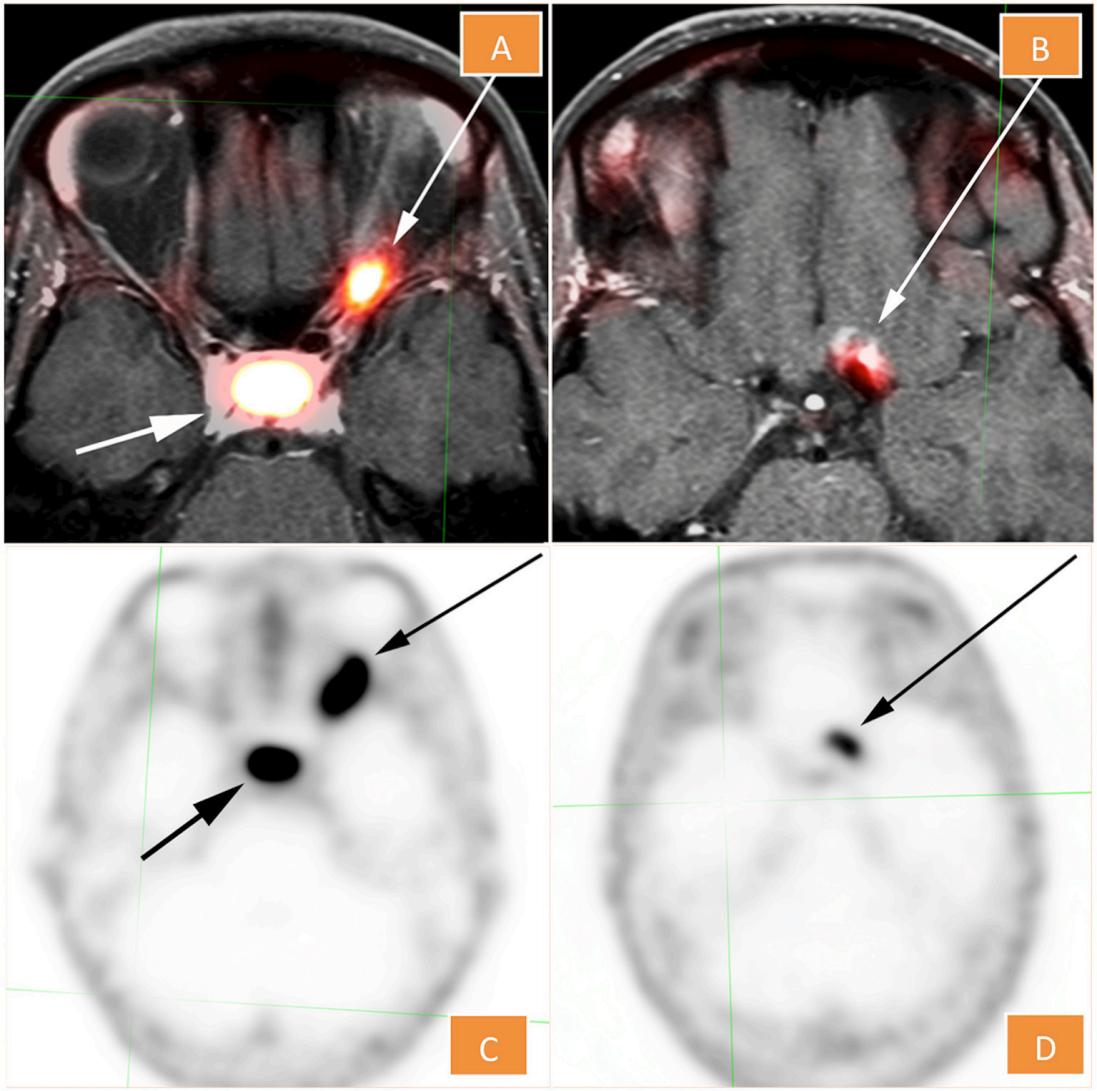
^68^Ga -DOTATATE PET **(C,D)**, fused with MRI in **(A,D)**. The lesion shows conspicuous diffuse radiotracer uptake and maximum standardized uptake value of 10.8. There is a nearby but separate focus that localizes to the left aspect of the planum sphenoidale **(B,D)**.

^68^Ga-DOTATATE PET/CT scan revealed an asymmetric fusiform enlargement of the left optic nerve with associated conspicuous diffuse radiotracer uptake and maximum standardized uptake value (SUV_max_) of 10.8. Portions of the lesion showed increased attenuation on non-contrast CT, suggesting calcification. There was a nearby but separate focus of activity more superoposteriorly, which localized to the left aspect of the planum sphenoidale (Figure 2). The combination of anatomic and metabolic findings was compatible with an optic sheath meningioma, as an optic nerve glioma, similarly to a pilocytic astrocytoma, would not be expected to demonstrate significant uptake on ^68^Ga-DOTATATE PET (5).

**Figure 3:**
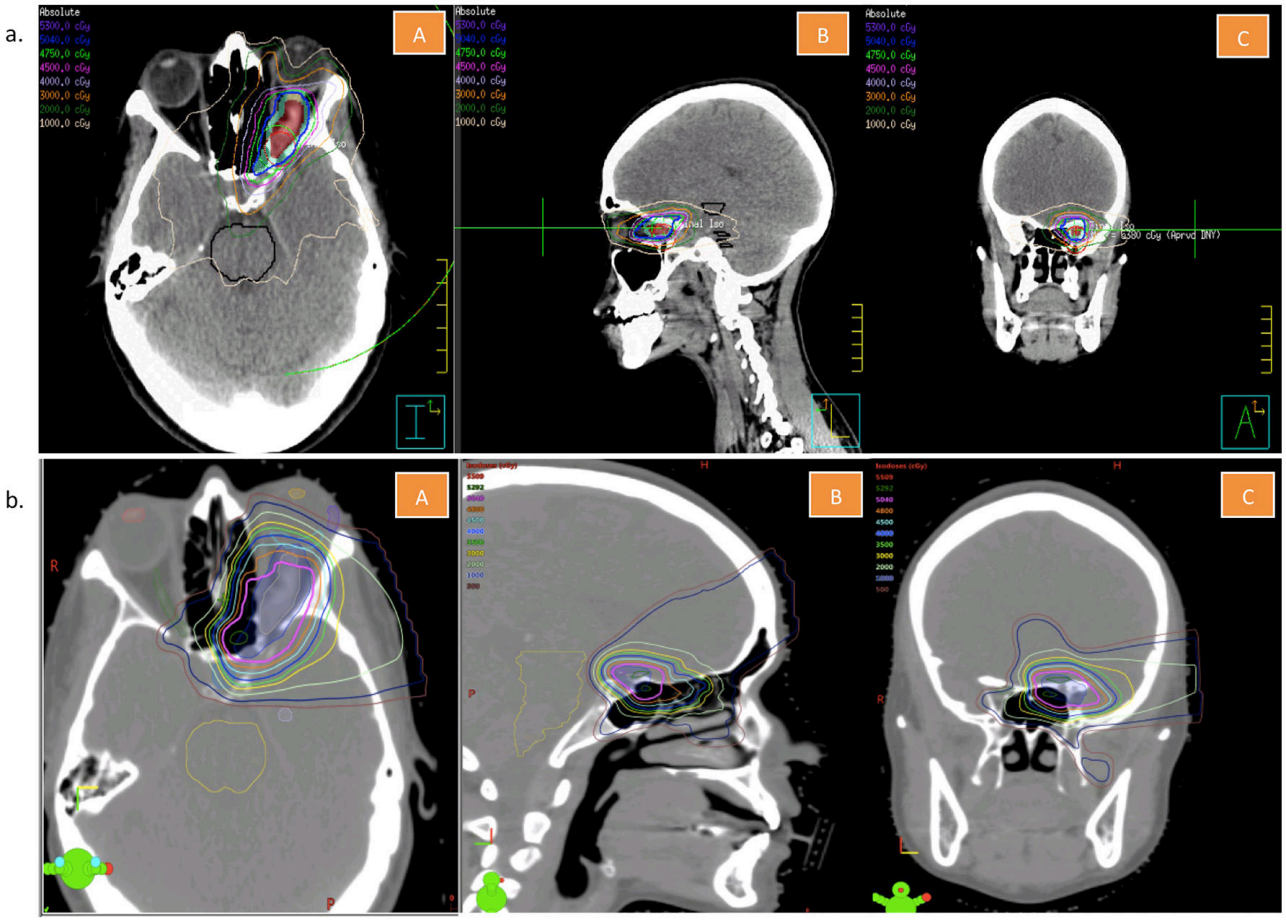
**(a)** Volumetric arc treatment plan of the patient described in this case report. This figure is showing representative cuts in the transverse (A), sagittal (B), and coronal (C) planes. **(b)** Tentative proton treatment plan of the patient described in this case report. This figure is showing representative cuts in the transverse (A), sagittal (B), and coronal (C) planes.

**Figure 4:**
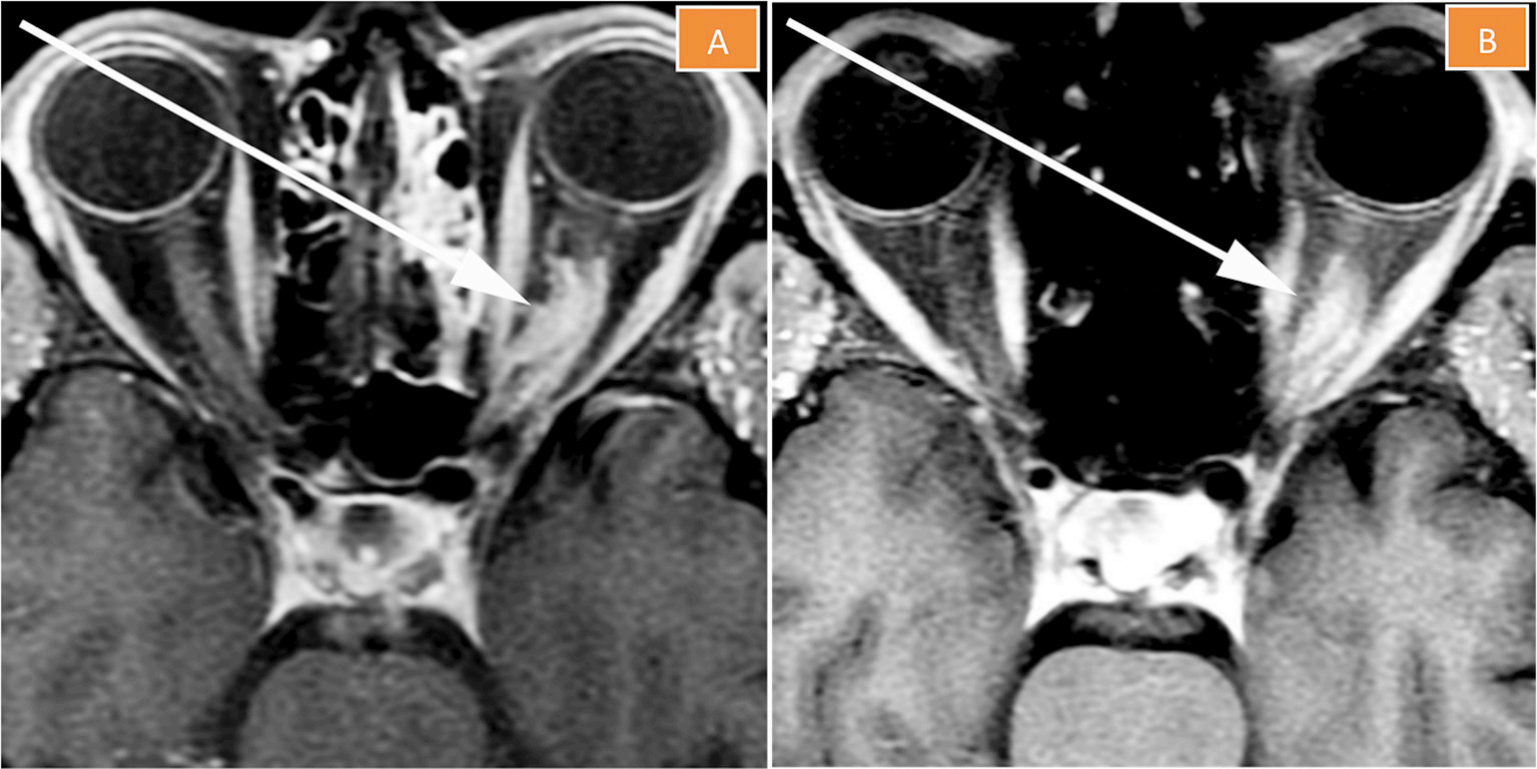
Magnetic resonance imaging of the left orbit 2 months **(A)** and 5 months **(B)** after treatment completion. The tumor is less enhancing than pre-treatment, its size is stable to slightly decreased, and “tram-tracking” is more apparent.

Based on this non-invasive diagnosis, volumetric-modulated arc therapy (VMAT) to a total dose of 50.4 Gray (Gy) in 28 fractions was delivered. A VMAT radiation plan (Figure 3A) was chosen over a proton therapy plan (Figure 3B) because of improved dose conformity and target coverage in the former. The patient had no major complications. She developed Common Terminology for Adverse events (CTCAE) Grade 1 fatigue and Grade 1 headaches during treatment. Five months after treatment completion, the patient had significant improvement in her left temporal hemianopia based on subjective report and on objective assessment through a formal visual field examination performed by her ophthalmologist. The meningioma on the 2- and 5-month follow-up MRIs was found to be less enhancing and its size was slightly decreased to stable (Figure 4), indicating good local control.

## Discussion

### ONSM: diagnosis and management

MRI and CT are currently the most commonly used imaging modalities in the workup of orbital space-occupying lesions (4). In a study by Sepahdari et al. evaluating 47 patients with orbital lesions, diffusion-weighted (DW) imaging was used to detect malignant lesions with a sensitivity of 63%, a specificity of up to 90%, and an accuracy of 81% (1). The incorporation of DWI to conventional MR imaging has helped improve radiological diagnoses and characterization of indeterminate orbital lesions. For the diagnosis of ONSM, MRI is considered the gold standard for determining a radiological diagnosis (6). Typically, ONSM displays intense enhancement with gadolinium on T1 fat-suppressed MR images of the orbit, and is surrounding a non-enhancing optic nerve (negative defect), creating the classic “tram-track” sign (7, 8). When the MR characteristics do not show all the hallmark features, the detection of calcification on CT can provide complementary imaging evidence to support the diagnosis of ONSM as calcification is better demonstrated on CT than MRI (9).

In certain instances, as in our case report, MRI and CT scan may not provide sufficient diagnostic information to differentiate ONSM from other optic tumors, such as optic pathway gliomas. An open orbital biopsy can provide definitive pathological diagnosis, but this procedure is associated with significant risks, including postoperative diplopia, orbital hemorrhage, and symblepharon (10). Albeit rare, these potential risks are compelling physicians to find an alternative non-invasive diagnostic modality for accurately identifying these tumors based on clinical information and imaging. We however acknowledge that, short of the gold standard of biopsy, the diagnosis of optic meningioma could not be confirmed with absolute certainty for the patient presented in this case report.

### Role of ^68^Ga-DOTATATE PET/CT and ^68^Ga-DOTATOC PET/CT in the diagnosis and management of ONSM

Meningioma cells have been shown to strongly express somatostatin receptor subtype 2 (SSTR2) (11), unlike pilocytic astrocytomas (including optic pathway gliomas) (5). This characteristic of meningioma cells is the basis of using molecular imaging with somatostatin receptor ligands, which is being proposed as an excellent tool to discriminate between these two medical entities.

The use of somatostatin receptor ligands in meningiomas, and even ONSM, is not novel. Some studies used ^111^Indium (In)-octreotide scintigraphy (Octreoscan^TM^) to differentiate meningiomas from other dural-based tumors, increasing the diagnostic accuracy of MRI (12), and others used this same technique, as a complement to serial MRIs, to follow-up ONSMs after treatment completion (13). However, Octreoscan^TM^ was found to lack specificity (12). More recently, two somatostatin analogs, DOTA (0)-D-Phe (1)-Tyr (3)-Octreotide (DOTATOC) and DOTATATE, were radiolabeled with ^68^Ga to be used in ^68^Ga-DOTATOC PET/CT and ^68^Ga-DOTATATE PET/CT. They have been found to have better lesion detectability and spatial resolution as compared to ^111^In-labeled peptides (14, 15), with significantly lower radiation exposure (16). Both DOTATOC and DOTATATE have been shown to have similar diagnostic accuracy in the setting of neuroendocrine tumors, with marginal superiority of DOTATOC, which shows higher tumor uptake (17, 18); that being said, they have not yet been compared in the setting of meningiomas.

A study by Rachinger et al. compared the diagnostic accuracy of ^68^Ga-DOTATATE PET/CT and MRI in 21 patients with primary or recurrent meningiomas. The former was shown to delineate meningioma from normal tissue better than the latter. Sensitivity to detect tumor tissue was 90.1% for ^68^Ga-DOTATATE PET/CT vs. 79% for MRI both in the primary and recurrent settings. Specificities (73 vs. 63%) and positive predictive values (89 vs. 84%) were, however, similar between the two imaging modalities (19).

A study by Klingenstein et al. on 13 patients with ambiguous lesions of the anterior optic pathway demonstrated excellent sensitivity (100%) and specificity (100%) for ^68^Ga-DOTATATE PET/CT. It allowed differentiation of meningioma (10 patients) from other entities (intracerebral metastasis, inflammatory connective tissue disease, and leukemic infiltrate), as meningiomas demonstrated high ^68^Ga-DOTATATE uptake (mean SUV_max_ = 14.3 ± 15.4), whereas the other three lesions did not (SUV_max_ = 2.1 ± 1.0) (20). The sample size of that study is too small to draw meaningful conclusions, but it is rather hypothesis-generating. In our case report, we are providing fused ^68^Ga-DOTATATE PET-MR imaging, not provided in Klingenstein's case series, thus allowing direct correlation between the anatomical and metabolic information from ^68^Ga-DOTATATE-PET and MRI, respectively.

Despite the fact that ONSM are indolent benign tumors, they can eventually lead to proptosis, progressive loss of visual acuity, and if left untreated, to blindness (3, 21). The optimal timing of intervention has not yet been fully elucidated, and observation with strict regular follow-up visits is considered a reasonable option as a first step if the patient has no or minimal visual symptoms (22, 23). Some experts have advocated that treatment becomes warranted when a new decline in acuity and/or visual fields has been documented (24), but the highly variable and unpredictable natural history of ONSM makes that strategy sometimes risky. Beyond its strength in diagnosis and discriminating between meningiomas and other tumors, ^68^Ga-DOTATATE PET/CT has shown the potential to predict tumor growth rate. Higher expression of SSTR2 resulting in higher SUV_max_ on ^68^Ga-DOTATATE PET imaging has been shown to predict for faster growth rates in World Health Organization (WHO) grade I and II meningiomas (25). As such, this imaging modality may provide guidance with respect to the initiation of therapy.

Treatment options include surgery in rare instances, and radiation therapy in most scenarios. Because of significant functional morbidity, surgery for ONSM has fallen out of favor, except in cases of rapid visual decline in which quick surgical decompression is needed, or in case of intracranial extension (26, 27). Stereotactic fractionated radiation therapy (RT) is now recommended as the treatment of choice in ONSM to stop tumor growth and preserve vision (28, 29). A retrospective study by Turbin et al. comparing observation, surgery, RT, or surgery and RT, showed that RT alone was associated with half the complication rates of surgery (33.3 vs. 66.7%), and with better visual preservation outcomes (30). RT for ONSM consists of 50.4–54 Gy at 1.8 Gy per fraction, typically using three-dimensional (3D) conformal plans, or intensity modulated radiation therapy (IMRT) plans (28, 31–34). Plans with VMAT as used in our case might allow for more conformal dose distribution as well as better tumor coverage and normal tissue sparing than conventional static field IMRT, while also allowing for reduced treatment delivery time (35). The use of proton therapy for ONSM is not well documented in the literature, but seems to offer comparable local control and vision preservation rates as photon radiation (28, 36). Proton plans may include the use of passive scatter with collimators or pencil beam scanning with intensity modulated proton therapy (IMPT). There may be range uncertainty with the use of protons, which can be an important consideration when evaluating treatment plans involving critical structures such as the optic nerves (37). Secondly, the relative biological effectiveness (RBE) may also be higher with protons, which is estimated to ~1.1 within a wide range of values (38). For the particular case and anatomy, our group favored VMAT/IMRT for the conformity and uniformity in tumor coverage it provided while maintaining the dose below organ-at-risk tolerance. In addition to its diagnostic utility, there is potential for ^68^Ga-DOTATOC PET to serve as a complementary modality to MRI (39, 40) in terms of tumor delineation for radiotherapy.

## Conclusion

Imaging with somatostatin receptor ligands seems to be a valuable tool in the non-invasive diagnosis of orbital space-occupying lesions. Further studies are needed to better establish the potential applications of ^68^Ga-DOTATATE PET/CT and ^68^Ga-DOTATOC PET/CT in the diagnosis, management, and follow-up of ONSMs, and in other intracranial meningioma types.

## Ethics statement

The patient described in this case report consented to have her case described in this manuscript. Written informed consent was obtained from the patient for publication of this case report. The patient's personal identifiers were not included in this manuscript.

## Author contributions

KA, CC, DY, and JJ contributed to the conception and design of the work. BC, MG, and JJ assembled and interpreted the patient's imaging data. KA and CC wrote the first draft of the manuscript. All authors contributed to manuscript revision, and provided approval for publication of the content.

### Conflict of interest statement

The authors declare that the research was conducted in the absence of any commercial or financial relationships that could be construed as a potential conflict of interest.
